# Paediatric acute liver failure: A prospective, nationwide, population‐based surveillance study in Germany

**DOI:** 10.1002/jpn3.70149

**Published:** 2025-07-07

**Authors:** Dominic Lenz, Muhammad Abdulaziz, Bianca Peters, Matias Wagner, Lea D. Schlieben, Victor M. Corman, Ulrich Baumann, Philip Bufler, Tal Dattner, Rainer Ganschow, Kristin Genzel, Nicole Hammann, Steffen Hartleif, Bianca Hegen, Stephan Henning, André Hoerning, Martin Jankofsky, Norman Junge, Simone Kathemann, Birgit Knoppke, Martina Kohl‐Sobania, Martin Laass, Elke Lainka, Eberhard Lurz, Michael Melter, Hanna Müller, Denisa Pilic, Markus Ries, Lisa Schiefele, Tobias Schwerd, Ekkehard Sturm, Mechtild Wegner, Michael S. Urschitz, Sven F. Garbade, Daniel Wenning, Christian Drosten, Alexander Fichtner, Stefan Kölker, Georg F. Hoffmann, Holger Prokisch, Christian Staufner

**Affiliations:** ^1^ Department I, Medical Faculty Heidelberg, Center for Pediatric and Adolescent Medicine, Division of Pediatric Neurology and Metabolic Medicine Heidelberg University Heidelberg Germany; ^2^ Clinic for Pediatrics I, University Children's Hospital Essen University Duisburg‐Essen Essen Germany; ^3^ School of Medicine, Institute of Human Genetics Technical University of Munich Munich Germany; ^4^ Helmholtz Zentrum München, Institute of Neurogenomics Computational Health Center Neuherberg Germany; ^5^ Institute of Virology Charité‐Universitätsmedizin Berlin, Corporate Member of Freie Universität Berlin, Humboldt‐Universität zu Berlin, Berlin Institute of Health Berlin Germany; ^6^ German Centre for Infection Research (Deutsches Zentrum für Infektionsforschung) Berlin Germany; ^7^ Department of Paediatric Kidney, Division for Paediatric Gastroenterology and Hepatology Liver and Metabolic Diseases, Hannover Medical School Hannover Germany; ^8^ Department of Pediatric Gastroenterology, Nephrology and Metabolic Diseases Charité Universitätsmedizin Berlin Berlin Germany; ^9^ Department of Pediatrics University Hospital Bonn Bonn Germany; ^10^ Pediatrics, HELIOS Klinikum Erfurt Erfurt Germany; ^11^ Paediatric Gastroenterology and Hepatology University Children's Hospital, Eberhard Karls University Tubingen Tubingen Germany; ^12^ Department of Pediatrics University Medical Center Hamburg‐Eppendorf Hamburg Germany; ^13^ Department of Pediatrics University Hospital Erlangen, Friedrich Alexander University of Erlangen‐Nürnberg Erlangen Germany; ^14^ Department of Pediatric Gastroenterology, Hepatology, and Transplant Medicine, Children's Hospital University Duisburg‐Essen Essen Germany; ^15^ KUNO University Children's Hospital, University Hospital Regensburg Regensburg Germany; ^16^ Department of General Pediatrics University Medical Center Schleswig‐Holstein Kiel Germany; ^17^ Children's Hospital, Medical Faculty Carl Gustav Carus Technische Universität Dresden Dresden Germany; ^18^ Department of Pediatrics, Division of Gastroenterology and Hepatology, Dr. von Hauner Children's Hospital University Hospital, LMU Munich Munich Germany; ^19^ Department of Pediatrics, Division of Neonatology and Pediatric Intensive Care University Hospital Marburg Baldingerstraße Marburg Germany; ^20^ Neonatology, University Children's Hospital, Eberhard Karls University Tubingen Tubingen Germany; ^21^ Department of Pediatrics and Adolescent Medicine, Division of Neonatology and Pediatrics University Medical Center Ulm, Ulm University Ulm Germany; ^22^ Department of Pediatrics University Hospital Greifswald Greifswald Germany; ^23^ German Paediatric Surveillance Unit (ESPED), Division of Paediatric Epidemiology, Institute of Medical Biostatistics, Epidemiology, and Informatics (IMBEI) University Medical Centre of the Johannes Gutenberg University Mainz Germany

**Keywords:** aetiology, ESPED, incidence, outcome, PALF

## Abstract

**Objectives:**

Paediatric acute liver failure (PALF) is a rare but life‐threatening condition, yet comprehensive epidemiological data in Germany are lacking. Our study aimed to systematically analyse incidence, aetiology, and outcome of PALF in Germany.

**Methods:**

In a nationwide, population‐based surveillance study, cases of PALF (defined following the PALF study group inclusion criteria) were queried from 2016 to 2018 through the German Paediatric Surveillance Unit (ESPED). Demographic, clinical, laboratory, therapeutic, and outcome data were collected and analysed. In case of unexplained aetiology, whole exome and virus sequencing was offered as a complementary diagnostic.

**Results:**

Over the 3‐year period, 148 cases were reported, yielding an estimated incidence of 3.7 per 1 million children per year. Neonates and infants were predominantly affected (45% of the cases); median age at PALF was 1.2 years (range: 0–17.9 years). Metabolic/genetic diseases were the most common cause (23%), followed by infectious causes (17%). The overall diagnostic yield was 73%, diagnosis remained unknown in 40 cases. Clinical outcome was age‐dependent: new‐borns showed a significant higher lethality (42%), followed by infants (29%), toddlers (15%), and school children (12%). Liver transplantation was reported in 22% of cases.

**Conclusions:**

This study provides comprehensive insights into PALF epidemiology in Germany. Metabolic/genetic causes and infectious diseases were most common. Advances in standardised diagnostic work‐up and genetic analysis have enhanced diagnostic yield, yet mortality remains substantial, particularly among neonates. Further research is warranted to improve diagnostic accuracy, therapeutic outcomes, and overall management of PALF.

## Abstract (highlights)

**What is Known:**

- Paediatric acute liver failure (PALF) is rare and life‐threatening.
- Little is known about epidemiological aspects of PALF in Germany and Europe.

**What is New:**

- Incidence of PALF in Germany: 3.7 per 1 million children per year with mainly children in their first year of life affected (49%).
- The overall diagnostic yield was 73%, metabolic/genetic causes are the leading aetiology (23%) followed by infectious causes (17%).
- Neonates have a significantly higher risk of a fatal outcome, while overall mortality was 18%.
- Whole exome sequencing revealed a genetic diagnosis in 13 of 47 unsolved cases.

## 1 INTRODUCTION

Paediatric acute liver failure (PALF) is a rare, life‐threatening event characterised by elevated liver enzymes and impaired liver function without known chronic liver disease.^1^ Understanding the aetiology is essential for specific therapies, prognosis and the decision regarding liver transplantation. In the US registry (*n* = 1144, PALF study group 1999–2014), paracetamol intoxication, metabolic diseases, and viral infections were common causes; however, in 43% of cases, the aetiology remained indeterminate.^2^, ^3^ Data on aetiology, incidence and outcome in Europe are sparse. In a recent survey among European expert centres, PALF was mainly caused by intoxications (23.5%), followed by genetic causes (18.8%).^4^ In Germany, data are limited to single centre experiences, with infectious and metabolic causes reported as most common. In line with the data from the PALF study group a proportion of 43% of the cases remained without a diagnosis.^5^ However, with the introduction of next‐generation sequencing into diagnostics, previously unknown causes of PALF have been identified in the last decade.^6^, ^7^, ^8^, ^9^, ^10^, ^11^, ^12^, ^13^ The implementation into routine care can significantly increase the diagnostic yield in this life‐threatening condition and change the aetiological landscape of PALF.^14^

To study this rare condition, the German Paediatric Surveillance Unit (ESPED) was contacted to include PALF into their ongoing nationwide surveillance. The ESPED is affiliated with the German Society for Paediatrics and Adolescent Medicine with the aim of collecting epidemiological data on rare paediatric diseases in Germany.^15^, ^16^ In 1995 and 1996, a first surveillance study on PALF was carried out and published in a conference abstract book. A total of 69 children were included with a median age of 1 year. Main aetiologies were infectious (45%), metabolic and toxic causes (each 16%) while 11 cases remained without diagnosis. Thirty‐one children died and twelve were transplanted. More details are not available from the abstract book.^17^

The aim of this study is to systematically analyse incidence, aetiology and outcome of PALF in Germany. By offering whole exome sequencing, virus sequencing and expanded metabolic studies on a research basis for unsolved cases, we aimed to explore the proportion of genetic diseases among indeterminate cases of PALF in Germany.

## 2 METHODS

### 2.1 Ethics statement

All procedures were in accordance with the ethical standards of the responsible committee on human experimentation and with the Helsinki Declaration of 1975, as revised in 2013. Informed consent to participate in the sequencing studies was obtained from all patients and/or from their parents in case of minor patients. The study was approved by the ethical committees of the Technical University Munich and the University Hospital Heidelberg. In accordance with the local ethics committee, no extra ethical approval was necessary for the anonymized surveillance study.

Population denominator data were extracted from the German Federal Statistics Office database (https://www.destatis.de/).

### 2.2 Study design and case definition

A prospective, nationwide surveillance study of PALF cases in Germany was carried out between January 1, 2016, and December 31, 2018. PALF was defined according to the inclusion criteria of the PALF study group in the United States as follows (all criteria had to be met):

1. Elevated serum concentrations of alanine transaminase (ALT), aspartate transaminase (AST) or bilirubin.
2. International normalised ratio (INR) ≥ 2 (or INR ≥ 1.5 if hepatic encephalopathy is present), not corrected by vitamin K.
3. No known chronic liver disease.

*Exclusion criteria:* Multiorgan failure following heart surgery or extracorporeal membrane oxygenation (due to difficulty to define liver‐based coagulopathy in this situation); history of solid organ or stem cell transplantation; acute trauma.

### 2.3 Data sources

PALF cases were identified through the ESPED.^18^ Data were actively collected on a monthly basis from all paediatric departments and departments of paediatric surgery.^18^ Physicians reporting a case of PALF were sent a standardised questionnaire to provide anonymized demographic data, clinical information, laboratory peak parameters, aetiology, therapy, and outcome (supplemental material: questionnaire version 1 and 2). Paediatric end‐stage liver disease (PELD) and model of end‐stage liver disease (MELD) scores were calculated.^19^ After 18 months, the questionnaire was adopted with a more precise differentiation in hepatic encephalopathy grades (in the early phase: grade 0–II combined). In case of unexplained aetiology, complementary metabolic and sequencing diagnostics was offered on an individual basis and outside the surveillance study. For metabolic diagnostics, acylcarnitine profile in dried blood spots, amino acids in plasma, organic acids in urine, galactose‐1‐phophate uridyltransferase activity and isoelectric focussing of transferrin in serum was measured in the study centre Heidelberg if not performed so far. To detect novel or previously unrecognised viruses, ribonucleid acid (RNA) and deoxyribonucleic acid (DNA) high‐throughput‐sequencing (HTS) and additional specific adeno‐associated virus 2 (AAV2)‐testing via polymerase chain reaction (PCR) of blood samples (Best et al. unpublished; available upon request) was performed at Charité Berlin from deoxyribonucleic acid (DNA) and ribonucleic acid (RNA) extracted (MagNA Pure 96 DNA and Viral NA Small Volume Kit; Roche Molecular Diagnostics) from blood during the PALF episode. HTS libraries were set up using Nextera® XT DNA Library Prep (Illumina) for DNA and KAPA RNA Hyper Prep kit (Roche) for RNA libraries and subsequently sequenced on Illumina NextSeq platform (Illumina) reaching a median of 3.8 (range 2.6–8.8) Million paired‐reads per sample Classification of reads was done using KRAKEN and DIAMOND algorithms as described elsewhere (REF: https://www.ncbi.nlm.nih.gov/pmc/articles/PMC10774810/). For genetic work‐up, whole‐exome sequencing (WES) at the Technical University of Munich was offered by the study team. Cases with indeterminate aetiology examined by WES were reported before.^9^ All PALF cases were independently reviewed and validated by a paediatric hepatologist and a specialist in paediatric metabolic medicine. Cases were re‐classified regarding their aetiology by the study team based on new findings (e.g., metabolic or genetic results) and results communicated with the caring physicians.

### 2.4 Statistical analysis

Incidence of definite cases with PALF were estimated from the number of children aged 0–18 years living in Germany during the study period. For comparative analysis *χ* ^2^ test and Mann–Whitney tests were performed. Tests were considered statistically significant with a *p*‐value < 0.05. For standardised Pearson residuals cut‐off was 1.96. For outcome prediction, additional area under the curve (AUC) values were calculated. Statistical analysis was done using ‘R’ version 4.0.4 and GraphPad Prism version 10.

## 3 RESULTS

### 3.1 Study cohort

**Figure 1:**
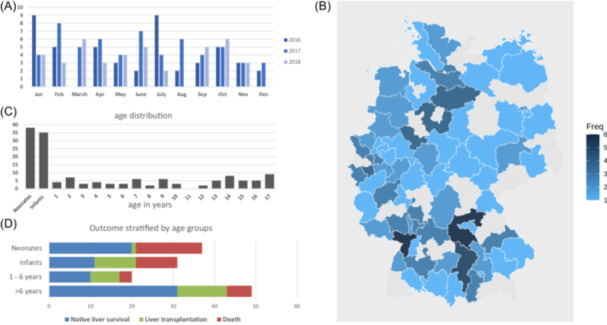
Epidemiological data. (A) Number of PALF cases per months throughout the 3 years of the study period. (B) Frequency of PALF cases within one postal code district regarding residency of affected individuals. (C) Age distribution within our cohort. (D) Outcome stratified by age groups. Death as endpoint: *χ* ^2^ *p*‐value: 0.0057; standardised Pearson residuals show more death than expected for neonates but less death than expected for children >6 years. Apr, April; Aug, August; Dec, December; Feb, February; Freq., frequency; Jan, January; Nov, November; Oct, October; PALF, paediatric acute liver failure; Sep, September.

During the 3‐year study period, 148 cases of paediatric ALF were reported, with minor differences of reporting frequency between years and months (Figure 1A). Given the average number of 13.5 million children between 0 and 18 years living in Germany per year during the study period,^20^ this results in an estimated incidence rate of 3.7 cases per 1 million children per year in Germany. There was inhomogeneous regional distribution of reported cases, with postal code areas without a single case within the study period (Figure 1B). Regarding sex, 59% of included individuals were male. The median age at PALF was 1.2 years with highest number of cases for neonates and infants (Figure 1C). The outcome at the time of reporting was age‐dependent: New‐borns showed the highest lethality, followed by infants, toddlers (1–6 years) and school children (>6 years). Mortality differed significantly in new‐borns and school‐aged children (Figure 1D).

### 3.2 Aetiology

Most frequent were metabolic/genetic causes (23%) followed by infectious causes (17%). Aetiology remained indeterminate in 27% of cases. Rate of indeterminate cases was highest in infants where 15 of 35 cases (43%) remained unsolved. Intoxication was common in school children. Most frequent single aetiologies were Wilson disease (*n* = 10), gestational alloimmune liver disease (*n* = 10), followed by mitochondrial disorders (*n* = 9) and autoimmune hepatitis (*n* = 9). Table 1 shows individual breakdown of aetiologies. In 47 unsolved cases, WES was performed unravelling the genetic cause in 13 of those (28%) with pathogenic variants in the following genes: *EIF2AK3, SLC25A15, TRMU, DLD, LARS1, ADK, DGUOK, NBAS*, and *MPV17*. Virologic and metabolic testing did reveal the aetiology in none of the indeterminate cases.

**Table 1:** Aetiologies of PALF.

Type of liver injury	Number of patients (in %)	Aetiologies	Neonatal (*n* = 38)	Infants (*n* = 35)	1–6 years (*n* = 21)	>6 years (*n* = 54)
Indeterminate	40 (27%)	‐	7	15	7	11
Metabolic/Genetic	34 (23%)	Wilson's disease (10), mitochondrial disorders (9), urea cycle disorders (4), infantile liver failures syndrome tpye 2 (*NBAS*) (3), tyrosinaemia type I (2), Wolcott‐Rallison syndrome (2), adenosine kinase deficiency (2), infantile liver failures syndrome tpye 1 (*LARS1*) (1), Progressive familial intrahepatic cholestasis (*ABCB11*) (1)	8	8	5	13
Infectious	25 (17%)	Influenza (5), enterovirus infection (5), adenovirus infection (4), cytomegalovirus infection (3), herpes simplex virus infection (3), sepsis (2), human herpesvirus 6 infection (2), parvovirus B19 infection (1)	6	6	5	8
Immunologic	21 (14%)	Gestational alloimmune liver disease (10), autoimmune hepatitis (9), haemophagocytic lymphohistiocytosis (2)	9	3	2	7
Toxic	12 (8%)	Amanita (3), paracetamol (3), DRESS by sulfasalazine (1), DRESS with no medication specified (1), fluconazole (1), valproate (1), methamphetamine (1)	0	1	2	9
Shock/multiorgan failure	12 (8%)		7	1	0	4
Others	4 (3%)	Biliary atresia (1), acute lymphatic leucemia (1), mesenchymal harmatoma (1), aplastic anaemia (1)	1	1	0	2

*Note*: Aetiology in all included cases throughout the whole study period, aetiologies and age group distribution of aetiology groups. Abbreviations: DRESS, drug rash with eosinophilia and systemic symptoms; PALF, paediatric acute liver failure.

### 3.3 Clinical and laboratory parameters

Ascites was reported in 60% of cases with no significant differences between aetiologic groups. In terms of laboratory chemistry, the children presented with a median ALT level of 1227 U/l (range: 18–11,363), an AST of 1538 U/l (range: 26–28,305), an INR of 2.88 (range: 1.5–20) and a bilirubin of 131.67 µmol/l (range: 2.63–1124.33). A trigger was reported in 116/144 cases; most common were febrile infections (*n* = 49), followed by medications and infections without fever (e.g., upper respiratory tract infections with only mildly elevated body temperature). Liver biopsy was performed in 43 individuals, 24 of these were during the PALF episode while 19 were performed at a later time point. Steatosis was present in 12 patients, fibrosis in 27 biopsies of which nine showed signs of liver cirrhosis. Necrosis was evident in 24 samples (Figure S1). The nine cases with cirrhosis included three neonates, three infants and three school children. In seven of the nine cases the aetiology was unravelled (Table S1).

In 80% of cases, extrahepatic involvement was reported, mainly affecting the neurologic system. Most patients presented without or with a mild (grade I and II) hepatic encephalopathy (HE, 71%), while moderate (grade III) and severe (grade IV) HE showed a similar frequency of 14% and 15% respectively. The second frequently reported neurological symptom was seizures, most frequent in the metabolic group and the group with indeterminate aetiology (Table 2).

**Table 2:** Clinical characteristics, therapeutic measures and outcome stratified by aetiology.

Clinical characteristics	Indeterminate (%) *n* = 40	Metabolic/genetic (%) *n* = 34	Infectious (%) *n* = 25	Immunologic (%) *n* = 21	Toxic (%) *n* = 12	Shock/MOF (%) *n* = 12	Others (%) *n* = 4	*χ* ^2^ *p*‐value
Ascites	29 (78)	18 (58)	15 (63)	8 (40)	6 (50)	5 (42)	3 (75)	0.0822
Seizures	8 (22)	6 (21)	3 (14)	0 (0)	1 (8)	1 (13)	0 (0)	0.7872
Hepatic encephalopathy grade III or IV	10 (36)	8 (26)	5 (25)	2 (12)	5 (45)	2 (40)	2 (50)	0.4312
Therapies								
Ventilation support	20 (51)	12 (39)	16 (64)	**6 (29)**	5 (42)	**12 (100)**	2 (50)	**0.0031**
Ciruclatory support	22 (56)	11 (35)	13 (52)	**4 (19)**	6 (50)	**12 (100)**	2 (50)	**0.0007**
Extracorporal renal replacement therapy	14 (36)	6 (19)	5 (21)	5 (24)	4 (33)	2 (18)	1 (25)	0.6843
Fresh frozen plasma	33 (36)	25 (78)	18 (72)	13 (62)	8 (67)	12 (100)	3 (75)	0.1399
Outcome								
Native liver survival	16 (42)	12 (40)	16 (70)	13 (65)	7 (64)	7 (58)	1 (33)	0.1962
Liver tranplantation	12 (32)	11 (37)	3 (13)	2 (10)	1 (9)	0 (0)	1 (33)	0.1002
Death	10 (26)	7 (23)	4 (17)	5 (25)	3 (27)	5 (42)	1 (33)	0.8515

*Note*: Ventilation support as endpoint: *χ* ^2^ *p* = 0.0031; standardised Pearson residuals shows less ventilation support in the immunologic group than expected but more ventilation support than expected for the Shock/MOF group. Circulatory support as endpoint: *χ* ^2^ *p* = 0.0007; standardised Pearson residuals shows less circulatory support in the immunologic group than expected but more circulatory support than expected for the Shock/MOF group. Clinical characteristics, therapeutic measures and outcome were equally distributed among the aetiology groups (*χ* ^2^‐test with *p*‐values > 0.05). Regarding outcome: data were not available for all patients. Abbreviation: MOF, multiorgan failure.

*Note*: Bold values are statistically significant.

### 3.4 Therapy

Nearly all patients received vitamin K (133/142) and fresh frozen plasma (FFP) (112/144). Half of the patients (51%) received respiratory support and 70/144 patients circulatory support with catecholamines. Extracorporeal renal replacement therapy (ERRT) was used in 37/106 cases. The use of ventilation and pressor support was significantly higher in patients with shock and lower in patients with immunologic aetiologies. Other therapeutic measures did not differ between aetiologic groups (Table 2).

### 3.5 Outcome

Data on outcome were available in 137 patients: 72 patients survived with their native liver, 30 underwent liver transplantation, and 35 patients had died by the time of reporting the questionnaire. Outcome did not differ between aetiologic groups (Table 2) and there was no difference regarding mortality (chi‐square test; *p*: 0.95) or native liver survival (chi square test; *p*: 0.51) when comparing low (up to one case per year) and high frequency areas (more than one case per year) based on their postal code areas. When outcome was stratified by age, neonates died in 44% of cases while liver transplantation was only performed in one neonatal case. Fatal outcome was less frequent in school children and more frequent in neonates (*χ* ^2^ test: *p*‐value: 0.0057) (Figure 1D). Furthermore, outcome was poor in the cases with cirrhosis detected in the biopsy (*n* = 9). Two children died and six underwent liver transplant (Table S1).

### 3.6 Predictors of outcome

Clinical and laboratory peak parameters and calculated PELD and MELD scores were assessed regarding outcome predictions. Therefore, the cohort was divided in two groups: individuals with native liver survival (*n* = 72) and the ones that underwent liver transplantation or died (*n* = 65). Mann–Whitney test revealed differences for ALT and AST levels, INR, bilirubin, ammonia, and albumin regarding laboratory peak parameters. Furthermore, PELD and MELD scores, need of pressor or ventilation support as well as ERRT differed between the groups while area under curve (AUC) values were low (Table 3).

**Table 3:** Clinical characteristics stratified by outcome category.

Parameter	Native liver survival *n* = 72	Death or transplanted *n* = 65	*p* value	Statistical test	ROC
	**Mean ± SD (median)**	**Mean ± SD (median)**			**AUC**
AST	3849 U/l ± 515 (2157 U/l)	2737 U/l ± 542 (1137 U/l)	**0.0145**	Mann–Whitney test	0.6217
ALT	2724 U/l ± 358 (1701 U/l)	1556 U/l ± 272 (546 U/l)	**0.0031**	Mann–Whitney test	0.647
INR	2.86 ± 0.17 (2.41)	4.25 ± 0.33 (3.80)	**<0.0001**	Mann–Whitney test	0.714
Bilirubin	158.5 µmol/l ± 17.2 (106.7 µmol/l)	243.4 µmol/l ± 27.6 (157.3 µmol/l)	**0.0087**	Mann–Whitney test	0.630
Creatinin	79.4 µmol/l ± 11.1 (46.9 µmol/l)	97.6 µmol/l ± 11.4 (61.0 µmol/l)	0.0663	Mann–Whitney test	0.593
Ammonia	98.2 µmol/l ± 7.4 (88.5 µmol/l)	279.8 µmol/l ± 100.3 (128.9 µmol/l)	**0.0006**	Mann–Whitney test	0.686
Lactate	6.1 µmol/l ± 0.58 (4.22 µmol/l)	8.3 µmol/l ± 0.83 (5.65 µmol/l)	**0.0551**	Mann–Whitney test	0.600
Albumin	27.4 g/l ± 0.94 (26.5 g/l)	23.8 g/l ± 0.89 (23.6 g/l)	0.0030	Mann–Whitney test	0.655
CK	3784 U/l ± 3003 (149 U/l)	1415 U/l ± 659 (209 U/l)	0.8852	Mann–Whitney test	0.509
Age	6.00 years ± 0.75 (2.95 years)	4.17 years ± 0.72 (0.49 years)	**0.1806**	Mann–Whitney test	0.567
PELD score	22.3 ± 1.3 (20.0)	33.8 ± 1.5 (34.0)	<0.0001	Mann–Whitney test	0.7720
MELD score	19.1 ± 1.1 (17.4)	27.4 ± 1.3 (26.8)	**<0.0001**	Mann–Whitney test	0.746
Hepatic encephalopathy grade III–IV	14/47 (30%)	20/46 (44%)	0.1705	*χ* ^2^ test	
Circulatory support	27/72 (38%)	42/63 (67%)	**0.0010**	*χ* ^2^ test	
Ventilation support	29/72 (40%)	42/63 (67%)	**0.0032**	*χ* ^2^ test	
ERRT	10/72 (14%)	27/63 (43%)	**0.0002**	*χ* ^2^ test	

*Note*: Standardised Pearson residuals were calculated in case of statistically significant *p*‐value in the *χ* ^2^ test. Cut‐off value for standardised Pearson residual was 1.96. Abbreviations: ALT, alanine transaminase; AST, aspartate transaminase; AUC, area under the curve; CK, creatine kinase; ERRT, extracorporeal renal replacement therapy; INR, international normalised ratio; MELD, model of end‐stage liver disease; PELD, paediatric end‐stage liver disease; ROC, receiver operating characteristic; SD, standard deviation.

*Note*: Bold values are statistically significant.

## 4 DISCUSSION

Our study reports 148 cases of PALF. The incidence of 3.7 cases per 1 million children per year is the first epidemiological assessment of this condition in Germany. Although this is likely an underestimate due to underreporting, PALF is not a rare,^21^ but an ultrarare condition.^22^ Its incidence is comparable with other rare conditions analysed by ESPED, for example, arterial ischaemic stroke with 4.1 cases per 1 million children per year.^23^

Interestingly, a first attempt to report on the German landscape of PALF in 1996 only resulted in 69 cases reported in a 2 years period.^17^ As an increase in incidence has not been reported elsewhere, the higher number of patients in our study points at an increased awareness and improved surveillance since then. One further aspect might have been the option of a free of charge WES, and extended metabolic and virologic analyses with the aim to solve the indeterminate cases. This is of major importance as clinical presentation often does not distinguish between aetiologies and unravelling the cause is crucial for treatment strategies.^5^, ^24^, ^25^, ^26^

We found 35 distinct aetiologies in our cohort with metabolic/genetic causes as the most important group. Compared to earlier studies, this is a relevant increase potentially due to the broader availability of diagnostic tools.^1^, ^3^, ^4^, ^5^ However, it was surprising that no single case of classical galactosemia presenting with ALF was documented during the study time. This might be due to the fact that the national new‐born screening program in Germany includes screening for classical galactosemia.

While classical diagnostic work‐up for metabolic diseases is fast and reveals treatable diagnoses, a relevant proportion of cases cannot be solved without genetic testing. In our study, a total of 47 unsolved cases underwent WES and 13 of those cases were solved. These cases were part of a larger WES study for PALF cases that even showed a diagnostic yield of 38%.^14^ And knowledge of the diagnosis facilitates specific therapies up to the decision regarding liver transplantation. Including the genetic approach, the diagnostic work‐up led to an ascertained diagnosis in 108 of the 148 reported cases (73%). Compared to earlier studies,^1^, ^3^, ^5^ this is a promising development using standardised diagnostic work‐ups together with novel diagnostic tools, contributing to a more specific patient management and potentially improved outcomes. However, in the group of infants beyond neonatal age the diagnostic yield remained in need of improvement with 43% of cases without diagnosis. We hypothesise that in this group yet undiscovered monogenic diseases may be underlying aetiologies in a considerable proportion, as the diagnostic yield of WES in indeterminate PALF has been reported to be specifically high in this age group.^24^, ^25^

Infections were the second most frequent aetiology in our cohort. While no hepatitis A–E infection was recorded, influenza, adenovirus, and enterovirus infections were reported followed by herpes simplex virus (HSV) and cytomegalovirus (CMV) infections. While all of them are known to cause PALF, the viral infection (e.g., CMV) might also be the second hit in a child with an undiscovered underlying genetic condition as it is known in genetic recurrent acute liver failure syndromes.^1^, ^13^ Further virologic work‐up with deep virus sequencing did not reveal more underlying infections, but samples were sparse as they need to be taken within the acute episode. With the background of the AAV2/Adenovirus associated non‐A–E‐hepatitis outbreak in 2022, our samples were reanalysed with an AAV2specific PCR based assay not revealing a single case with AAV2/Adenovirus.^27^, ^28^ However, advanced virologic diagnostics including next‐generation sequencing remain an important tool in unsolved hepatitis/PALF cases. Additionally, the role of infections as a trigger to PALF—reported in 49 cases—should prompt further investigation in this field.

Among the 14% of immunologic causes, there was an unexplained high incidence of gestational alloimmune liver disease cases with 10 cases reported within the study period. There is no explanation for the high number of cases and future studies are necessary to explore this further. A toxic cause was reported in 8% of our cases with three cases of paracetamol intoxication. This contrast to data in the United States (16% of cases in the PALF study group)^1^, ^2^, ^3^ might be due to the fact, that in Germany paracetamol is not sold as an over‐the‐counter drug in high quantities. A further relevant fraction was due to shock and multiorgan failure; however, this may still be an underestimate, as liver injury with coagulopathy in the context of multiorgan failure may not be titled PALF in many centres. Indeed, shock and multiorgan failure was reported from single centres only. Finally, the inclusion of a case of biliary atresia is questionable as this represents a setting of an acute on chronic liver injury.

The clinical presentation in our cohort did not differ between aetiologic groups. Main reported clinical symptoms were ascites, seizures and severe hepatic encephalopathy. Regarding therapeutic interventions, nearly all patients received vitamin K and a majority of patients received FFP in the therapeutic course although there is no standard recommendation for the application of FFP in PALF.^2^ The use of FFP before invasive procedures such as central venous access, venous catheter placement for extracorporeal renal replacement therapy (ERRT), liver biopsies or intracerebral pressure (ICP) monitoring could explain the high percentage in our cohort. The necessity of an intensive care setup is strengthened by the fact that half of the patients required ventilation and pressor support. The presence of cirrhosis in the biopsy of nine, mainly metabolic/genetic cases, indicates a chronic severe remodelling as a sign of an unrecognised underlying chronic liver disease and therefore an acute on chronic liver injury. Those cases showed poor native liver survival in only one case and a high rate of liver transplantation performed in 6/9. This reflects the progressed liver remodelling. However, there might be more hidden acute on chronic liver failure cases as biopsies during PALF episodes are of questionable value and their indication should be questioned thoroughly due to the potential high complication rate. In this study, no additional information on biopsy techniques, complications and contribution to establishing a diagnosis were retrieved to further evaluate benefit and harm of biopsies in PALF.

While there was no difference in relation to outcome between aetiology groups including the group of indeterminate cases, the study delineates notable differences based on clinical and laboratory parameters: High activity levels of ALT or AST were associated with a better outcome. On the other hand, high INR, ammonia and bilirubin levels as well as a low albumin predict unfavourable outcome, in line with previous reports.^5^, ^29^ MELD and PELD scores, developed for end‐stage liver disease could to some extent predict the outcome of PALF in our study. This is potentially mainly based on the high INR and bilirubin levels being already a single, well‐known predictor.

Clinical parameters including necessity of therapeutic interventions as ERRT or ventilation and pressor support were found to be associated with a negative clinical outcome as reported in previous studies.^30^ However, the use of single (peak) values to predict outcome are controversially discussed in recent years and new efforts are made to look at dynamic data collected over several days throughout the PALF episode that might be more adequate in this clinical condition.^31^, ^32^, ^33^ Furthermore, AUC values did not indicate promising stand‐alone parameters among those for reliable prediction of outcome. Moreover, the incorporation of both death and liver transplantation into one outcome group holds further limitations as the natural course of the disease is interrupted by liver transplantation. While outcome was better in older children (>6 years) and worse in neonates, an unpaired *t*‐test for the whole cohort was not significant, only showing a trend (*p* = 0.0814). Another limitation inherent in our study is the reliance on data assessed at one time point only that lies in the nature of the surveillance unit; however, in specific cases important follow‐up data as outcome were reported at a later time point by the surveillance unit on request of the study team.

## 5 CONCLUSION

In conclusion, this nationwide surveillance study on PALF in Germany provides the first published epidemiologic data with an incidence rate of 3.7 cases per 1 million children per year, mainly affecting children in their first year of life. This dynamic and life‐threatening clinical condition needs a high awareness in all paediatric hospitals to early recognise this condition and to initiate a recommended referral of the patient to a centre of excellence. Laboratory parameters might guide therapeutic decisions regarding prognosis of spontaneous recovery. Finally, monogenic diseases were the leading cause of PALF; hence, we recommend exome or genome sequencing to be applied early in the diagnostic course of PALF.

## CONFLICT OF INTEREST STATEMENT

The authors declare no conflicts of interest.

## Supporting information


**Supplemental Figure S1. Liver biopsies**. In 43 cases a liver biopsy was performed either within the Paediatric acute liver failure (PALF) episode (n = 24) or after the PALF episode. Most common finding was group cell necrosis in 15 cases followed by small droplet steatosis. PALF, paediatric acute liver failure.

Supporting information.

**Supplemental material: questionnaire version 1.** Standardized questionnaire to provide anonymized demographic data, clinical information, laboratory peak parameters, aetiology, therapy and outcome used in the first 18 months of the study.

**Supplemental material: questionnaire version 2.** Standardized questionnaire to provide anonymized demographic data, clinical information, laboratory peak parameters, aetiology, therapy and outcome used after the first 18 months of the study with a more precise, detailed item for hepatic encephalopathy.
