# Supplementary material for: Paediatric acute liver failure: A prospective, nationwide, population‐based surveillance study in Germany
Source: J Pediatr Gastroenterol Nutr. 2025 Jul 7;81(3):653–62. doi: 10.1002/jpn3.70149 (PMC12408955; doi:10.1002/jpn3.70149)
Supplement: Supplementary file 1 — Supplemental Figure S1. Liver biopsies. In 43 cases a liver biopsy was performed either within the Paediatric acute liver failure (PALF) episode (n = 24) or after the PALF episode. Most common finding was group cell necrosis in 15 cases followed by small droplet steatosis. PALF, paediatric acute liver failure. [file JPN3-81-653-s003.pptx]

## Slide 1
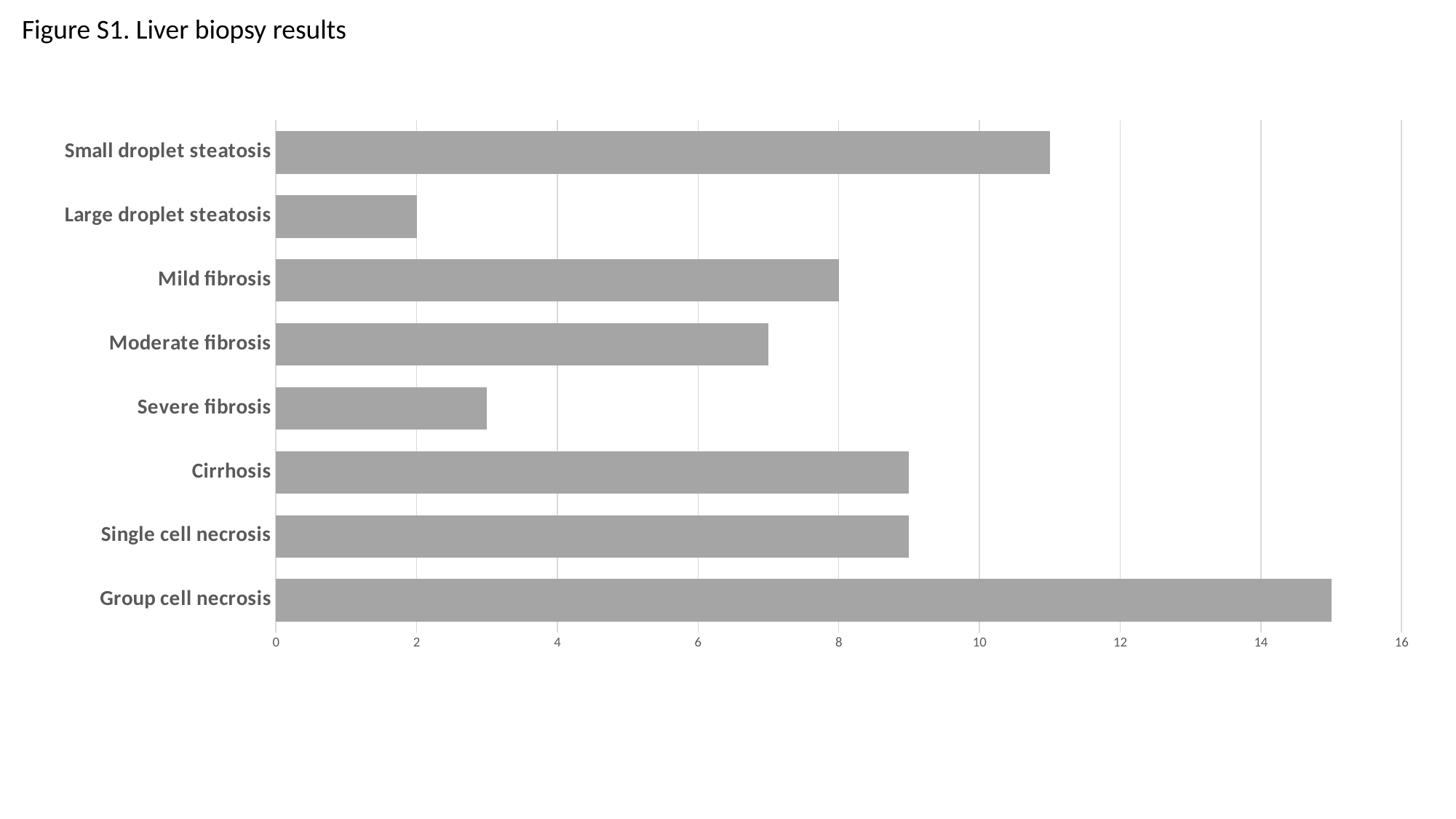

### Chart
| Category | |
|---|---|
| Group cell necrosis | 15.0 |
| Single cell necrosis | 9.0 |
| Cirrhosis | 9.0 |
| Severe fibrosis | 3.0 |
| Moderate fibrosis | 7.0 |
| Mild fibrosis | 8.0 |
| Large droplet steatosis | 2.0 |
| Small droplet steatosis | 11.0 |Figure S1. Liver biopsy results
