# Supplementary material for: Paediatric acute liver failure: A prospective, nationwide, population‐based surveillance study in Germany
Source: J Pediatr Gastroenterol Nutr. 2025 Jul 7;81(3):653–62. doi: 10.1002/jpn3.70149 (PMC12408955; doi:10.1002/jpn3.70149)
Supplement: Supplementary file 2 — Supporting information. [file JPN3-81-653-s002.docx]

| **Age group** | **Number of patients** | **Aetiologies** | **Outcome** |
| --- | --- | --- | --- |
| **Neonatal** | 3 | Adenosinkinase deficiency | Death |
|  |  | Gestational alloimmune liver disease | Liver transplantation |
|  |  | Inderterminate aetiology | Native liver survival |
| **Infants** | 3 | Gestational alloimmune liver disease | Death |
|  |  | Tyrosinemia type I | Liver transplantation |
|  |  | Inderterminate aetiology | Liver transplantation |
| **1 - 6 years** | 0 |  |  |
|  |  |  |  |
|  |  |  |  |
| **> 6 years** | 3 | Wilsons's disease | Liver transplantation |
|  |  | Wilsons's disease | Liver transplantation |
|  |  | Wilsons's disease | Liver transplantation |

**Table S1. Patients with cirrhosis in the liver biopsy.** Age distribution, aetiologies and outcome.
