# Supplementary material for: Paediatric acute liver failure: A prospective, nationwide, population‐based surveillance study in Germany
Source: J Pediatr Gastroenterol Nutr. 2025 Jul 7;81(3):653–62. doi: 10.1002/jpn3.70149 (PMC12408955; doi:10.1002/jpn3.70149)
Supplement: Supplementary file 3 — Supplemental material: questionnaire version 1. Standardized questionnaire to provide anonymized demographic data, clinical information, laboratory peak parameters, aetiology, therapy and outcome used in the first 18 months of the study. [file JPN3-81-653-s004.docx]

#
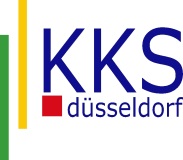

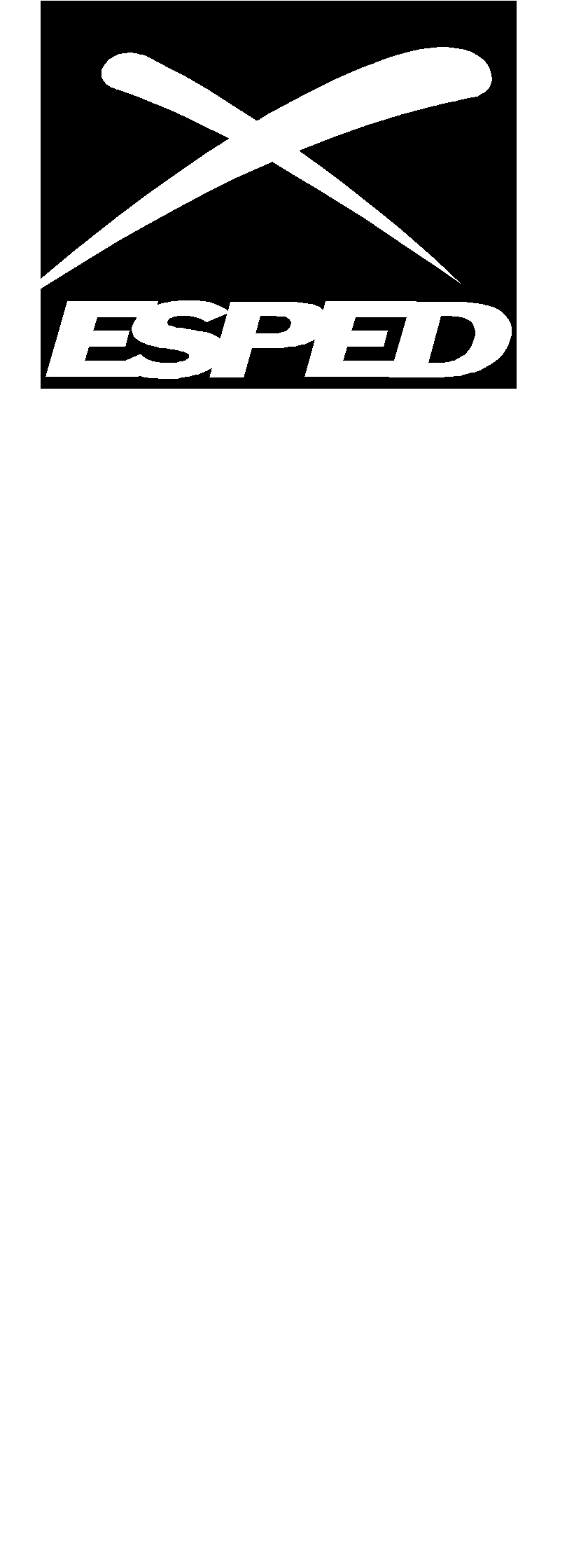
Erhebungseinheit für seltene pädiatrische Erkrankungen

in Deutschland

Forschungsstelle für pädiatrische Epidemiologie bei der Deutschen Gesellschaft für Kinder- und Jugendmedizin e.V.

**Koordinierungszentrum für**

**Klinische Studien (KKS) der**

**Heinrich-Heine-Universität Düsseldorf**

Leiter: Dr. Jürgen Grebe

**ESPED-Geschäftsstelle**

Prof. Dr. Ulrich Göbel

Prof. Dr. Rüdiger von Kries

Koordination: Dipl.Ing. (FH) Beate Heinrich

Tel.: 0211/81-16263

Fax: 0211/81-16262

beate.heinrich@med.uni-duesseldorf.de

http://www.esped.uni-duesseldorf.de

Postanschrift: Postfach 10 22 44

40013 Düsseldorf

Hausanschrift: Moorenstraße 5

40225 Düsseldorf

*2012 -*

*20 Jahre ESPED !*

**Wissenschaftlicher Beirat**

Prof. Dr. Ursula Felderhoff-Müser (Vorsitzende)

Zentrum für Kinder- und Jugendmedizin

Universitätsklinikum Essen

Dr. jur. Roman Christoffel

Rechtsanwalt

Düsseldorf

Prof. em. Dr. Guido Giani

Institut für Biometrie und Epidemiologie

Deutsches Diabetes-Zentrum Düsseldorf

Prof. em. Dr. Ulrich Göbel

Heinrich-Heine-Universität Düsseldorf

Dr. Jürgen Grebe

Koordinierungszentrum für Klinische Studien

Heinrich-Heine-Universität Düsseldorf

Prof. Dr. Rüdiger von Kries, MSc

Institut für Soziale Pädiatrie und Jugendmedizin

Epidemiologie im Kindes- und Jugendalter

Ludwig-Maximilians-Universität München

Prof. Dr. Ertan Mayatepek

Zentrum für Kinder- und Jugendmedizin

Universitätsklinikum Düsseldorf

Priv.-Doz. Dr. Annette Queißer-Wahrendorf

Zentrum für Kinder- und Jugendmedizin

Johannes Gutenberg-Universität Mainz

Priv.-Doz. Dr. Ole Wichmann

Infektionsepidemiologie

Robert Koch-Institut Berlin

Prof. Dr. Stefan Wirth

Zentrum für Kinder- und Jugendmedizin

HELIOS Klinikum Wuppertal

Universität Witten-Herdecke

ESPED, Arbeitsgruppe am KKS der Universität, Postfach 10 22 44, 40013 Düsseldorf

«ANREDE»

«TITEL» «NAME»

«KLINIK»

«ABT»

«STR»

«PLZ» «ORT»

27. September 2016

**Akutes Leberversagen im Kindes- und Jugendalter**

Meldemonat: **«MONAT»**

Fall-Nr.: «LNR»

Sehr geehrte«ANSUPP1» «ANSUPP2» «ANTITEL» «ANNAME»,

vielen Dank für die Meldung eines Ihrer Patienten mit akutem Leberversagen. Bitte senden Sie den Erhebungsbogen recht zeitnah im beigefügten, für Sie portofreien Briefumschlag zurück.

Die Studienziele sind die Inzidenzermittlung des akuten Leberversagens, die Erfassung der ätiologischen Verteilung sowie die phänotypische Charakterisierung der einzelnen ätiologisch differenzierten Entitäten anhand klinischer, laborchemischer und histologischer Parameter.

**Optionales Angebot: Bei ungeklärtem akutem Leberversagen** bietet das Studienzentrum Heidelberg in Kooperation mit dem Institut für Humangenetik der TU München und dem Institut für Virologie am Universitätsklinikum Bonn folgende **weiterführende Diagnostik** an, **für Sie kostenfrei**:

- Komplettierende metabolische Diagnostik
- Exom-Sequenzierung
- Detektion bisher unerkannter und unbekannter Viren

Für Details zu Einverständniserklärung, Probenmaterial und Versand siehe beiliegendes Informationsblatt oder unter <http://www.esped.uni-duesseldorf.de/esped/erkrankungen>. Bei Rückfragen können Sie sich gerne jederzeit an Herrn Dr. Staufner (Tel. 06221-5638377, christian.staufner@med.uni-heidelberg.de), Herrn Dr. Lenz (Tel. 06221-5635788, dominic.lenz@med.uni-heidelberg.de) oder das ESPED-Büro wenden.

Vielen Dank für die Unterstützung und freundliche Grüße,

gez. Dr. med. Christian Staufner Beate Heinrich

(ESPED-Studienleiter „Akutes Leberversagen“) (ESPED-Koordina­torin)

Akutes Leberversagen im Kindes- und Jugendalter

**Für Ihre Unterlagen!**

Meldemonat: «MONAT»

Fall-Nr.: «LNR»

Familienname:

Vorname:

Geb.-Datum:

Pat.Nr.:


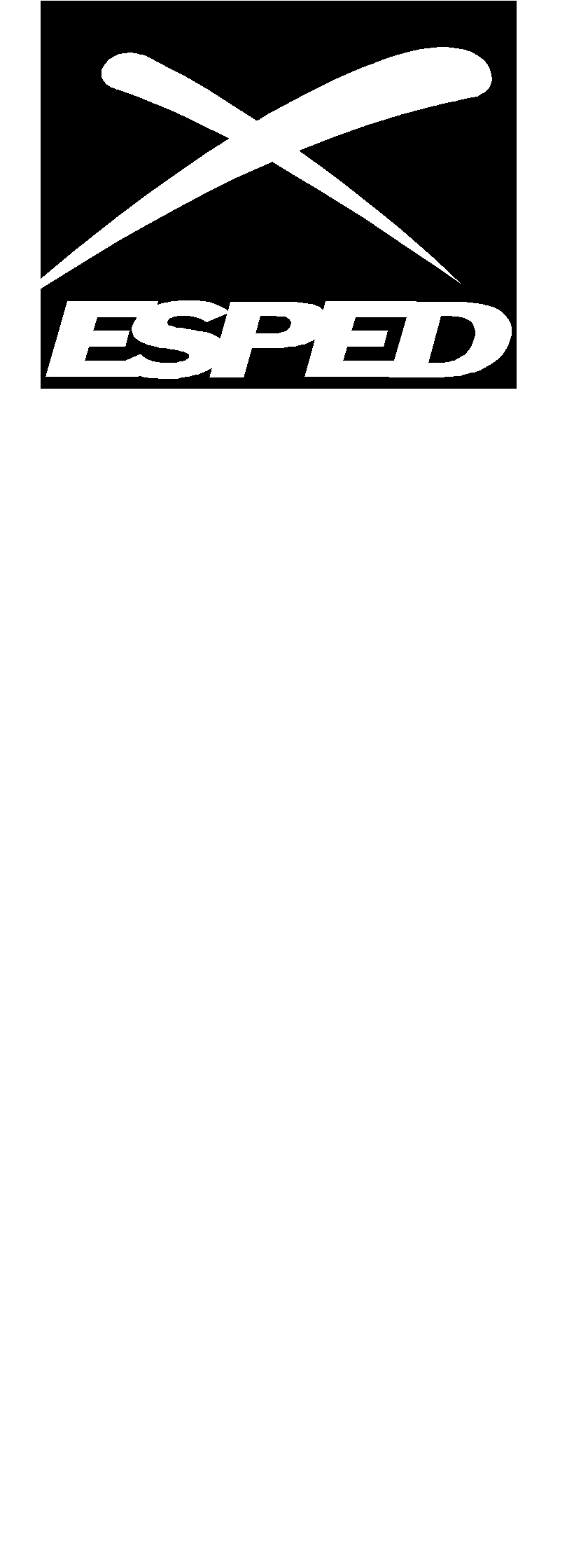
Erhebungseinheit für seltene pädiatrische Erkrankungen


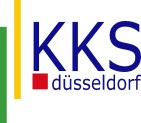
in Deutschland

Forschungsstelle für pädiatrische Epidemiologie bei der Deutschen Gesellschaft für Kinder- und Jugendmedizin e.V.

**Akutes Leberversagen im Kindes- und Jugendalter***

|  | ESPED  Arbeitsgruppe am KKS der  Heinrich-Heine-Universität Düsseldorf  z.Hd. Frau Heinrich  Postfach 10 22 44  40013 Düsseldorf | ⇦ | ***Rücksendung an nebenstehende Adresse erbeten!*** | |
| --- | --- | --- | --- | --- |
|  |  |  | IDNO:  LNR:  Meldemonat:  ESPED-Eingang: | *«IDNO»*  **«LNR»**  «MONAT» |

Berichtende Kinderklinik: Ansprechpartner für Rückfragen (*Bitte Druckbuchstaben*):

*«KLINIK» ..............................................................................................*

*«ABT»*

*«STR»* Telefon: .....................................….......................................

*«PLZ» «ORT»*

E-mail: .....................................……………...........................

#### Patienten-Datenblatt (verbleibt in der ESPED-Geschäftsstelle!)

#### Patientendaten

Geburtsdatum (Tag/Monat/Jahr): |___|___|________|

Geschlecht: 🞏 weiblich

🞏 männlich

PLZ Wohnort: |___|___|___|___|___|

Stationäre Aufnahme (Tag/Monat/Jahr): |___|___|________|

*in Zusammenarbeit mit der Gesellschaft für Pädiatrische Gastroenterologie und Ernährung e.V. (GPGE) und der Arbeitsgemeinschaft für Pädiatrische Stoffwechselstörungen (APS)

**ESPED: Akutes Leberversagen im Kindes- und Jugendalter** *Fallnummer:* ***«Lnr»***

**Klinik-Fragebogen** Seite 1/2

| *Wird von ESPED ausgefüllt:*  **1. Basisdaten:** Geburtsdatum: ____ /________ Geschlecht: weiblich / männlich  PLZ Wohnort: \|___\|___\|___\| |  |
| --- | --- |

| **2. Charakterisierung des akuten Leberversagens (ALV)** |  |
| --- | --- |
| **Alter bei Diagnosestellung:** …… Jahre …… Monate *Bei Alter <1 Monat*: …… Tage |  |
| **Vorangegangenes Ereignis / Trigger:**  Infekt mit Fieber  Infekt ohne Fieber  Medikamente (letzte 4 Wochen, auch Dauertherapie)  Welche: …………………………………………..………………  …………………………………………..………………..…………  Anderes: …………………………………………….…………….  Kein Trigger ersichtlich | Unbekannt |
| **Hepatische Enzephalopathie:**  Keine oder gering (0-II)  Somnolenz (III)  Koma (IV)  **Hepatomegalie:**  Ja  Nein  **Aszites:**  Ja  Nein | Unbekannt  Unbekannt  Unbekannt |
| **Laborparameter (maximale Werte im ALV, im Blut gemessen):** |  |
| ALAT (GPT) …………………………… U/L |  |
| ASAT (GOT) …………………………… U/L |  |
| Creatinkinase (CK) …………………………… U/L |  |
| Gamma-Glutamyltransferase (GGT) …………………………… U/L |  |
| Alkalische Phosphatase (AP) …………………………… U/L |  |
| Gesamt Bilirubin ……………………………  µmol/L  mg/dL |  |
| Direktes Bilirubin ……………………………  µmol/L  mg/dL |  |
| Kreatinin ……………………………  µmol/L  mg/dL |  |
| Albumin (minimaler Wert) …………………………… g/L |  |
| Cholinesterase (CHE) (minimaler Wert) …………………………… U/L |  |
| Glukose (minimaler Wert) ……………………………  mmol/L  mg/dL |  |
| Laktat ……………………………  mmol/L  mg/dL |  |
| Ammoniak …………………………… ☐ µmol/L  µg/dL |  |
| Quick-Wert (minimaler Wert) …………………………… % (nach Gabe von Vitamin K i.v.) |  |
| International Normalized Ratio (INR) …………………………… (n/a) (nach Gabe von Vitamin K i.v.) |  |
|  |  |
| **Leber-Biopsie erfolgt:**  Ja, im ALV  Ja, zeitl. Abstand zu ALV: ………… Tage  Nein  Fibrose /Zirrhose (Einteilung in Analogie an Desmet VJ et al., Hepatology 1994)  Keine Fibrose (0)  Portale Fibrose (1)  Periportale/septenbildende Fibrose (2)  Septenbildende Fibrose mit Architekturstörung (3)  Zirrhose (4)  Steatose  Keine Steatose  Kleintropfige Steatose  Grobtropfige Steatose  Zelluntergänge  Keine Zelluntergänge  Einzelzellnekrosen  Gruppenzellnekrosen  Weitere Auffälligkeiten: …………………………………………………………..……………..…………………… | Unbekannt |
| **3. Therapie, Outcome und Komorbiditäten** | |
| **Therapie:** Vitamin K i.v.  Ja  Nein  Fresh frozen plasma (FFP)  Ja  Nein  Darmdekontamination  Ja  Nein  Beatmung  Ja  Nein  Kreislaufunterstützung (katecholaminpflichtig)  Ja  Nein | Unbekannt  Unbekannt  Unbekannt  Unbekannt  Unbekannt |

**ESPED: Akutes Leberversagen im Kindes- und Jugendalter** *Fallnummer:* ***«Lnr»***

**Klinik-Fragebogen** Seite 2/2

| Dialyse-Verfahren  Ja  Nein  Hämofiltration  Hämodialyse  Plasmapherese  Andere: ………………………………………………..…..…  Hochdosiert Glucose i.v.*  Ja  Nein  Lipide i.v.  Ja  Nein  Carnitin i.v.  Ja  Nein  Weitere Therapien  Ja  Nein  Welche: ………………….…………………………..……….....  ……………….……………………….…………………….…….  * Säuglinge ca. 15g/kg/d; Kinder ca. 8-10g/kg/d | Unbekannt  Unbekannt  Unbekannt  Unbekannt  Unbekannt |
| --- | --- |
| **Outcome (nach ALV):**  Restitutio ad integrum  Chronische Hepatopathie  Transplantation  Tod |  |
| **Weitere klinische Symptomatik:**  Asymptomatisch  Ja  Nein  Mentale Entwicklungsstörung  Ja  Nein  Motorische Entwicklungsstörung  Ja  Nein  Muskuläre Hypotonie  Ja  Nein  Epilepsie  Ja  Nein  ADHS  Ja  Nein  Kleinwuchs (Körpergröße < 2SDS/3. Pz)  Ja  Nein  Dystrophie (Körpergewicht <2SDS/3. Pz)  Ja  Nein  Intrauterine Wachstumsretardierung  Ja  Nein  Andere Komorbiditäten  Ja  Nein  Welche: ………………………………………..……….……....  ……………………………………………………………………..  …………………………………………………………………….. | Unbekannt  Unbekannt  Unbekannt  Unbekannt  Unbekannt  Unbekannt  Unbekannt  Unbekannt  Unbekannt  Unbekannt |
| Vormals Episoden erhöhter  Ja  Nein  Transaminasen/ Bilirubin ohne ALV: Manifestationsalter der einzelnen Episoden:  ……………………………………………………………….……  Rezidivierendes akutes  Ja  Nein  Leberversagen: Manifestationsalter der einzelnen Episoden: ……………………………………………………………….…… | Unbekannt  Unbekannt |
| **4. Ätiologie des akuten Leberversagens** |  |
| Ätiologie **sicher** **geklärt**  Ursache: ……………………………………..…………………...………………………….……….….  Sicherung der Diagnose mittels: ………………………………………………………......................  Ätiologie **nicht sicher** **geklärt** (klinischer Verdacht ohne positive Bestätigungsdiagnostik) **^§^**  Verdachtsdiagnose: ……………………………………………………………………………...……..  Ätiologie **ungeklärt ^§^**  **^§^** siehe optionales Angebot zur kostenfreien weiterführenden metabolischen, virologischen und genetischen Diagnostik. Details auf ESPED-Website http://www.esped.uni-duesseldorf.de/esped/erkrankungen. |  |

**Vielen Dank für Ihre Mitarbeit!**


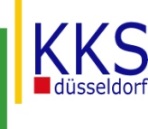

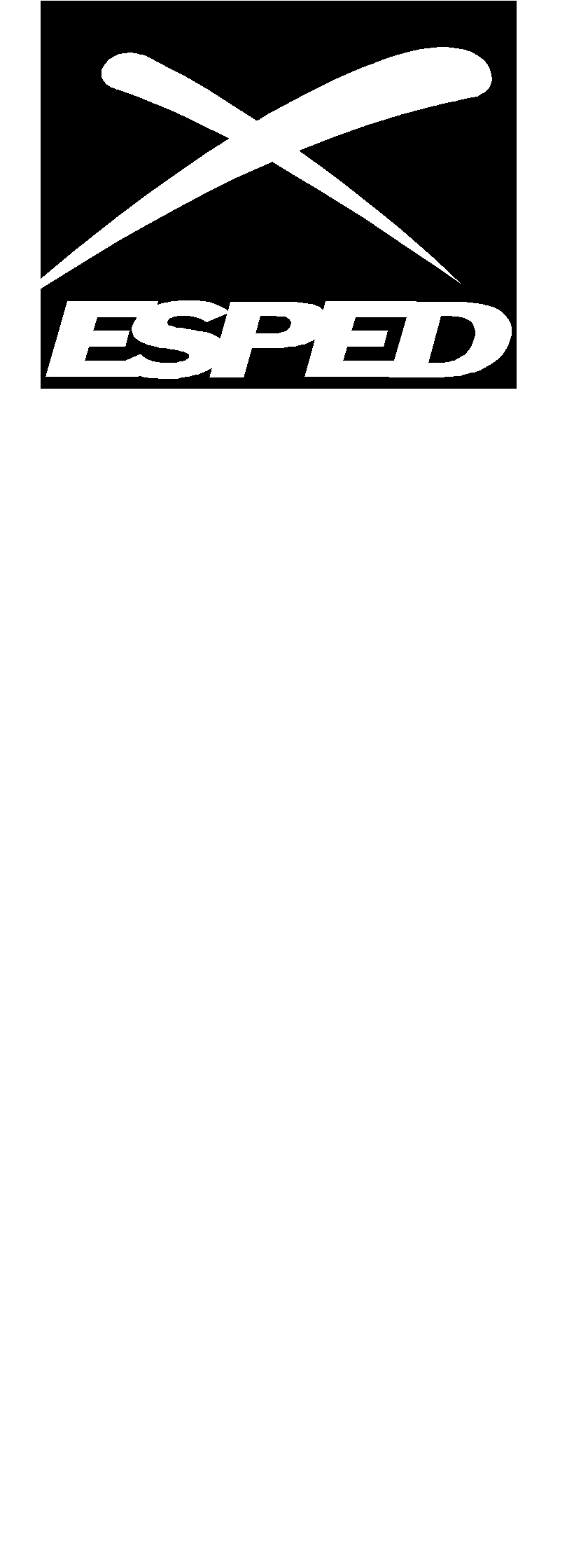


# Erhebungseinheit für seltene pädiatrische

# Erkrankungen in Deutschland

**Akutes Leberversagen im Kindes- und Jugendalter**

In Zusammenarbeit mit der Gesellschaft für Pädiatrische Gastroenterologie und Ernährung e.V. (GPGE)
 und der Arbeitsgemeinschaft für Pädiatrische Stoffwechselstörungen (APS)

**Bei ALV ungeklärter/nicht sicher geklärter Ätiologie** bietet das Studienzentrum Heidelberg folgende Diagnostik an (für Sie **kostenfrei**):

- - Komplettierende metabolische Diagnostik
  - Exom-Sequenzierung
  - Detektion bisher unerkannter und unbekannter Viren

Patienteninformation und Einverständniserklärung siehe ESPED Homepage
(<http://www.esped.uni-duesseldorf.de/esped/erkrankungen>) oder auf Anfrage.

**Probenmaterial**

| **möglichst in der Krise abgenommen  (Stoffwechsel und Virologie)** | **unabhängig der Krise  (Genetik)** |
| --- | --- |
| 0,5-1 ml **Serum** (1-2ml Serum-Vollblut) | 2-5ml **EDTA**-**Vollblut** (separates Röhrchen) |
| 1-1,5 ml **EDTA**-**Plasma** (2-3ml EDTA-Vollblut) |  |
| **Trockenblutkarte** |  |
| 5-10ml **Urin** |  |

**Versand**

- Serum, EDTA-Plasma (jeweils nicht Vollblut!) und Urin auf Trockeneis
  + Trockenblutkarte und EDTA-Vollblut (Genetik) bei Raumtemperatur **ODER**
- alle Proben per Express bei Raumtemperatur (direkt nach Abnahme)

**Versandadresse (Proben und Einverständniserklärung)**

Dietmar-Hopp-Stoffwechselzentrum

Stoffwechsellabor

Stichwort „ESPED-ALV-Studie“

Im Neuenheimer Feld 669

69120 Heidelberg

**Kontakt**

Dr. Christian Staufner ([Christian.Staufner@med.uni-heidelberg.de](mailto:Christian.Staufner@med.uni-heidelberg.de), Tel. 06221-5638377)

Dr. Dominic Lenz (Dominic.Lenz@med.uni-heidelberg.de, Tel. 06221-5635788)
